# Epigenetic impacts of stress priming of the neuroinflammatory response to sarin surrogate in mice: a model of Gulf War illness

**DOI:** 10.1186/s12974-018-1113-9

**Published:** 2018-03-17

**Authors:** David G. Ashbrook, Benjamin Hing, Lindsay T. Michalovicz, Kimberly A. Kelly, Julie V. Miller, Wilfred C. de Vega, Diane B. Miller, Gordon Broderick, James P. O’Callaghan, Patrick O. McGowan

**Affiliations:** 1 Department of Biological Sciences and Center for Environmental Epigenetics and Development and Department of Cell and Systems Biology, University of Toronto, Scarborough campus, Toronto, ON Canada; 2 Present address: Department of Genetics, Genomics and Informatics, University of Tennessee Health Science Center, Memphis, TN 38103 USA; 3 Department of Psychology, University of Toronto, Toronto, ON Canada; 4 Department of Physiology, University of Toronto, Toronto, ON Canada; 5 Present address: Department of Psychiatry, Medical Laboratories, The University of Iowa, Iowa City, Iowa 52246 USA; 6 CDC-NIOSH, 1095 Willowdale Road, Morgantown, WV 26505 USA; 7 Center for Clinical Systems Biology, Rochester General Hospital Research Institute, Rochester, NY USA

**Keywords:** Gulf War illness, Corticosterone, CORT, Diisopropyl fluorophosphate, DFP, Acetylcholinesterase inhibitors, AChE, Transcriptomics, Epigenetics

## Abstract

**Background:**

Gulf War illness (GWI) is an archetypal, medically unexplained, chronic condition characterised by persistent sickness behaviour and neuroimmune and neuroinflammatory components. An estimated 25–32% of the over 900,000 veterans of the 1991 Gulf War fulfil the requirements of a GWI diagnosis. It has been hypothesised that the high physical and psychological stress of combat may have increased vulnerability to irreversible acetylcholinesterase (AChE) inhibitors leading to a priming of the neuroimmune system. A number of studies have linked high levels of psychophysiological stress and toxicant exposures to epigenetic modifications that regulate gene expression. Recent research in a mouse model of GWI has shown that pre-exposure with the stress hormone corticosterone (CORT) causes an increase in expression of specific chemokines and cytokines in response to diisopropyl fluorophosphate (DFP), a sarin surrogate and irreversible AChE inhibitor.

**Methods:**

C57BL/6J mice were exposed to CORT for 4 days, and exposed to DFP on day 5, before sacrifice 6 h later. The transcriptome was examined using RNA-seq, and the epigenome was examined using reduced representation bisulfite sequencing and H3K27ac ChIP-seq.

**Results:**

We show transcriptional, histone modification (H3K27ac) and DNA methylation changes in genes related to the immune and neuronal system, potentially relevant to neuroinflammatory and cognitive symptoms of GWI. Further evidence suggests altered proportions of myelinating oligodendrocytes in the frontal cortex, perhaps connected to white matter deficits seen in GWI sufferers.

**Conclusions:**

Our findings may reflect the early changes which occurred in GWI veterans, and we observe alterations in several pathways altered in GWI sufferers. These close links to changes seen in veterans with GWI indicates that this model reflects the environmental exposures related to GWI and may provide a model for biomarker development and testing future treatments.

**Electronic supplementary material:**

The online version of this article (10.1186/s12974-018-1113-9) contains supplementary material, which is available to authorized users.

## Background

A coalition of 34 countries deployed approximately 956,600 troops during the 1990–1991 Gulf War [1], with the majority, ~ 700,000, from the USA. An estimated 25–32% of these veterans fulfil the requirements of a Gulf War illness (GWI) diagnosis [2]. GWI is an archetypal, medically unexplained, chronic condition characterised by persistent sickness behaviour, with neuroimmune and neuroinflammatory components.

Symptoms of GWI include fatigue, musculoskeletal pain, cognitive dysfunction, chemical sensitivities, loss of memory and sleep disruption, which can be characterised as ‘sickness behaviour’ [3, 4]. ‘Sickness behaviour’ is normally a result of inflammatory response to illness or injury, which usually resolves itself over time after the initial insult is removed. Symptoms were reported within 6 months of the conflict [5–7].

Although the exact cause of GWI is still unknown, there is a consensus that exposure to environmental toxins is the most likely cause [8]. GWI symptoms are highly heterogeneous, and specific symptoms may be related to specific experiences: for example, Gulf War veterans exposed to nerve agents or oil well fires are at increased risk of brain cancer compared to other Gulf War veterans [9].

A leading hypothesis for the cause of GWI is that the high physical and psychological stress of combat interacted with exposure to acetylcholinesterase (AChE) inhibitors [1, 4, 10–19]. Military personnel were exposed to a number of AChE inhibitors [1, 20], including pyridostigmine bromide (PB), a reversible AChE inhibitor, as a prophylactic against nerve agents; sarin, soman, and related nerve agents, irreversible AChE inhibitors, which combatants were inadvertently exposed to after demolition of Iraqi supply depots, such as at Khamisiyah; organophosphate pesticides, irreversible AChE inhibitors, which were widely used to prevent pest-borne diseases and irritation [20]; permethrin, an insecticide which may inhibit AChE [10, 21]; and DEET, an insect repellent and a weak AChE inhibitor which may enhance the activity of other AChE inhibitors [22]. For example, an estimated 95,000 deployed personnel were exposed to the plume from the Khamisiyah demolition, and approximately 250,000 may have been exposed to low levels of nerve agents during aerial bombardments earlier in the conflict [23]. Further, the number of nerve agent alarms heard is correlated with risk for GWI [24]. Accumulating research has indicated that deleterious health effects of exposures to psychophysiological stress [25, 26] and environmental toxicants [27, 28] involve epigenetic modifications that affect transcriptional regulation.

The overall objective of this study was to examine genome-wide epigenetic transcriptional modifications in the brain using an established mouse model of GWI [4, 12, 15, 16]. Our previous research demonstrates that effects on neuroinflammatory pathways occur shortly after initial exposures. For example, pre-exposure with the stress hormone corticosterone (CORT) causes an increase in expression of specific chemokines and cytokines in response to diisopropyl fluorophosphate (DFP) [4], an irreversible acetylcholinesterase inhibitor [29] used here as a sarin surrogate. This corresponds well with the work in GWI study participants [30–37], which have shown immunological abnormalities, and a recent paper [38], which has shown specific immune-related biomarkers for GWI veterans. We hypothesized that epigenetic and transcriptomic changes upon initial exposures would identify gene pathways linked to poor health outcomes in GWI.

## Methods

### Animals

Adult male C57Bl/6J mice were purchased from Jackson Laboratory (Bar Harbor, ME, USA). A total of 79 animals were used for the analyses presented here. All procedures were performed under protocols approved by the Institutional Animal Care and Use Committee of the Centers for Disease Control and Prevention, National Institute for Occupational Safety and Health and the US Army Medical Research and Materiel Command Animal Care and Use Review Office. The animal facility was certified by AAALAC International. Upon receipt, the mice were housed individually in a temperature (21 ± 1 °C) and humidity-controlled (50 ± 10%) colony room maintained under filtered positive-pressure ventilation on a 12-h light/12-h dark cycle beginning at 06:00 h. The plastic tub cages were 46 × 25 × 15 cm; cage bedding consisted of heat-treated pine shavings spread at a depth of approximately 4 cm. Teklad 7913 irradiated NIH-31 modified 6% rodent chow, and water were available *ad libitum*.

### Dosing

**Fig. 1:**
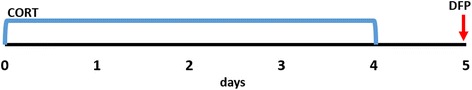
Overview of exposure timeline. CORT + DFP exposed animals were given CORT in the drinking water for 4 days and injected with DFP on the 5th day, before being culled 6 h later

The dosing paradigm is presented in Fig. 1. Mice were given CORT in the drinking water (200 mg/L in 0.6% EtOH) for 4 days. This CORT regimen is known to be immunosuppressive as evidenced by decreased thymus weight [39]; thymus and spleen weights were confirmed to be decreased (> 20%) in similarly exposed animals [4, 15]. On day 5, mice were given a single intraperitoneal injection of either DFP (4 mg/kg) or saline (0.9%).

Thus, there were four exposure groups: (1) saline: vehicle for 4 days, then saline injection on day 5; (2) CORT: CORT for 4 days with a saline injection on day 5;( 3) DFP: vehicle for 4 days with DFP injection on day 5; and (4) CORT + DFP: CORT for 4 days with a DFP injection on day 5.

### Brain dissection and tissue preparation

Mice were killed by decapitation and the brains rapidly removed. The frontal cortex, consisting of the anterior portion of the cortex [4], and total hippocampus were dissected free-hand on a thermoelectric cold platform (Model TCP-2; Aldrich Chemical Co., Milwaukee, WI, USA) and immediately frozen at − 80 °C.

### Differential gene expression

Frontal cortex mRNA-seq data was generated on the Illumina HiSeq 2000 by Q Squared Solutions Expression Analysis LLC (Morrisville, NC, USA), paired-end, with a read length of 100 bp (four samples of saline, CORT and DFP, five samples of CORT + DFP). Hippocampus mRNA-seq data was generated by Sickkids (Toronto, Ontario). Sequencing was carried out on the HiSeq 2500, paired-end, with a read length of 125 bp (*n* = 4 for all groups).

Fastq files were trimmed to remove adaptors and low-quality reads (*q* < 30) using TrimGalore version 0.4.1 [40], around Cutadapt [41] (version 1.9.1). The pre-processed reads were examined using FastQC [42].

Trimmed files were aligned with the STAR aligner [43] (version 2.5.2a) in a two-pass mode. The GENCODE GRCm38.p4 assembly (mm10) and annotations were obtained from the GENCODE website [44, 45] and used throughout. For the frontal cortex, there was an average of 33,547,161 reads per sample and 98.2% average mapped reads. For the hippocampus, there was an average of 35,429,647 reads per sample and 98.2% average mapped reads.

The resultant Bam files were analysed using the GenomicAlignments [46] and DEseq2 [47] (version 1.10.1) R packages, using the DEseq2 standard pipeline. A recent comparison study has identified this as an appropriate tool to use with replicates and when relatively large biological effects are expected [48]. Briefly, DESeq2 first fits a generalized linear model for each gene, with read counts modelled as a negative binomial distribution. An empirical Bayes approach is used for shrinkage of dispersion estimation, and the Wald test is used for significance testing, which is then adjusted for multiple corrections using the Benjamini and Hochberg method [49].

Samples were checked for similarity using Poisson dissimilarity matrix [50] with the R package PoiClaClu 1.0.2 and visualised with pheatmap 1.0.8.

### Cell proportions

The R Bioconductor package DeconRNASeq [51] was used to estimate the proportion of different cell types within the sample from the RNA-seq data. Data enriched for specific CNS cell types were downloaded from the Gene Expression Omnibus (GEO) [52, 53], Series GSE52564, which contains data from the *Mus musculus* cerebral cortex [54], to use as a reference of cell-type-specific gene expression. This RNA-seq data was trimmed and aligned and gene expression quantified as above. An expression signature for each of six cell types (astrocytes, neurons, oligodendrocyte precursor cells (OPC), myelinating oligodendrocytes (MO), microglia and endothelial cells) was obtained by finding those genes with a five-fold difference in expression in one cell type, compared to each of the others.

Using these expression signatures, the proportion of astrocytes, neurons, oligodendrocyte precursor cells (OPC), myelinating oligodendrocytes (MO), microglia and endothelial cells were estimated for each of the 17 cortex samples and 16 hippocampus samples using DeconRNASeq [51]. DeconRNASeq is based on a linear model of a sum of pure tissue or cell-type-specific reads of all cell types, weighted by the respective cell-type proportions. To estimate the proportions of known tissue types in a sample, DeconRNASeq solves a non-negative least-squares constraint problem with quadratic programming to obtain the globally optimal solution for estimated fractions. It is accurate down to cell types making up only ~ 2% of the total cell populations [51].

### DNA methylation modifications

DNA was extracted using the E.Z.N.A Tissue DNA Kit (VWR-Omega Bio-Tek). Bisulfite conversion was carried out using the Qiagen Epitect Fast Bisulfite Conversion Kit, and library preparation was performed using the Ovation NuGen RRBS Kit. Reduced representation bisulfite sequencing (RRBS) was carried out by the Princess Margaret Genomics Centre, part of the University Health Network, Toronto, on a NextSeq 500, using a single end, 70 base read length and multiplexed at 9–10 samples per flowcell. Samples for the cortex were 6 saline, 6 CORT, 6 DFP and 8 CORT + DFP, and for the hippocampus, 4 saline, 5 CORT, 3 DFP and 8 CORT + DFP.

RRBS fastq files were trimmed to remove adaptors and low-quality reads (*q* < 30) using TrimGalore version 0.4.1 [40] around Cutadapt [41] (version 1.9.1). Trimmed files were then aligned to the GENCODE GRCm38.p4 (mm10) assembly, using Bismark (v0.16.0) [55] wrapped around bowtie2 (version 2.2.6) [56]. For the frontal cortex, the average reads per sample was 34,962,881 with 57% mapping efficiency, and for the hippocampus, the average reads per sample was 37,512,807, 63.6% mapping efficiency.

The resultant bam files were analysed with MethPipe (3.4.2) [57, 58], using the suggested methods. The bisulfite conversion rate was > 98.9 for all samples. The methylation level for every cytosine site at single-base resolution is estimated as a probability based on the ratio of methylated to total reads mapped to that loci. Differential methylation was calculated by beta-binomial regression, with all batches and exposures included as factors, and CORT + DFP exposure set as the test factor. We examined both differentially methylated cytosines (DMCs) and differentially methylated regions (DMRs).

### Chromatin accessibility

H3K27ac is a mark of active enhancers, strongly suggesting that genes with differential enrichment of H3K27ac will be differentially expressed [59, 60]. Native chromatin immunoprecipitation sequencing (ChIP-seq) using an MNase digestion and alignment to the mm10 genome using Burrows–Wheeler Alignment was carried out by the Genome Sciences Centre, BC Cancer Agency. Samples were sequenced single end, 75 base read length on a Hiseq 2500 platform. The average read per sample was 131,727,189 with 98.9% average mapped reads. There were four samples per group, with each sample having immunoprecipitated and input DNA sequenced.

PePr (Python) and diffReps (Perl) packages were used for ChIP-seq analysis, as a recent paper by Steinhauser et al. [61] suggested that both are good tools when biological replicates are available. The results from each were compared and analysed to provide a conservative list of sites showing differential enrichment.

PePr (1.1.14) [62] was run according to the authors’ suggested pipeline. PePr used a sliding window approach, shifting all reads toward their 3′ direction by half of the empirically estimated DNA fragment length and estimating window width based on the average width of the top pre-candidate peaks. The genome was then divided into consecutive widths that overlap by 50%, and the number of reads within each window was counted. This read count was then normalised based on total read count among ChIP and control sample and the relative average peak heights among ChIP samples. Read counts were modeled across replicates and between groups with a local negative binomial model. Genomic regions with less variable read counts across replicates were ranked more favourably than regions with greater variability, thus prioritizing consistently enriched regions [62]. Narrow peaks were assumed, in line with previous literature on H3K27ac (e.g. [63]).

diffReps (1.55.6) [64] was run according to the authors’ suggested pipeline. Bam files first had to be converted to bed files, using Bedtools (v2.26.0) [65]. Unlike PePr, diffReps used a set window size of 1000 bp for narrow peaks with a step size of 100 bp. The genome was pre-screened to remove regions with low read count to improve power and decrease computational time. Normalisation was carried out using the read count for a particular window over read count across all samples. An exact negative binomial test was used for differential analysis, which used biological replicates. *p* values were adjusted by the Benjamini-Hochberg method [49]. Peaks were annotated to genes using region_analysis [64].

### Identifying genes unique to the CORT + DFP exposure

For several computational tools, e.g. DESeq2, PePr and diffReps, only direct comparisons between any two exposure groups (1v1) could be made, rather than the multifactorial comparisons available for differential DNA methylation modifications with MethPipe (RRBS). Therefore, in these cases, a series of 1v1 comparisons were made to conservatively estimate which genes were differentially expressed (RNA-seq) or enriched for H3K27ac (ChIP-seq). To use the RNA-seq data as an example, the 1v1 comparisons were carried out as:

1. Genes differentially expressed between CORT and CORT + DFP and then include only the subset of genes which were not differentially expressed between saline and DFP.
  - Changes between CORT and CORT + DFP may be due to DFP or the combination of CORT + DFP; removing those differentially expressed between saline and DFP removes those which are due to DFP alone
2. Genes differentially expressed between DFP and CORT + DFP and then include only the subset of genes which were not differentially expressed between saline and CORT.
  - Changes between DFP and CORT + DFP may be due to CORT or the combination of CORT + DFP; removing those differentially expressed between saline and CORT removes those which are due to CORT alone
3. Intersect of the genes which appear in both list 1 and list 2
  - Both should be genes differentially expressed due to the combined CORT + DFP exposure
4. Those genes which appear in list 3 and are differentially expressed between saline and CORT + DFP
  - Final check, as they should be different in the CORT + DFP exposure vs saline

This provides a list of changes unique to the combined CORT + DFP exposure, which are not seen in either CORT or DFP exposure alone. Note that this is conservative, as genes with low but significant differential expression in either CORT or DFP alone, but a larger change in the combined CORT + DFP exposure, will be lost.

### Enrichment analysis

For the sets of significant genes identified by RNA-seq, RRBS and ChIP-seq, as well as those genes that were significant in two or more of these analyses, gene set enrichment analyses were carried out. The R package clusterProfiler 3.4.4 [66] was used for Gene Ontology Biological Process (GO BP) [67, 68] and KEGG pathway [69, 70] enrichment analysis, with *p* and *q* value cutoffs of ≤ 0.05. Reactome pathway analyses were carried out using the ReactomePA 1.20.2 R package [71], with a *p* value cutoff of ≤ 0.05. All three packages reference the latest versions of their respective databases. These significantly enriched annotations were then visualised with the ‘enrichMap’ function of DOSE 3.2.0 R package [72], with parameters altered to aid legibility with different numbers of enriched annotations.

### Overlap between gene sets

The overlap between gene sets was visualised using the UpSetR R package [73]. This provides similar information to a Venn diagram, but in a way which makes proportions clear. Significance of overlap was determined by permutation analysis: random gene sets of the same size as our observed gene sets were taken from the same annotations, and the overlap between the two random gene sets was recorded. This was repeated 1,000,000 times, and the number of occurrences of an overlap equal to or larger than our observed overlap was divided by 1,000,000 to give an empirical *p* value.

## Results

### Differential gene expression

Samples were clustered using a Poisson dissimilarity matrix to determine if samples from the same exposure group showed similar expression profiles. As can be seen in Additional file 1: Figure S1 and Additional file 2: Figure S2, the samples largely clustered by exposure group. The only sample that appeared to be out of place was a CORT + DFP sample in the frontal cortex that appeared intermediate between CORT and DFP alone.

**Fig. 2:**
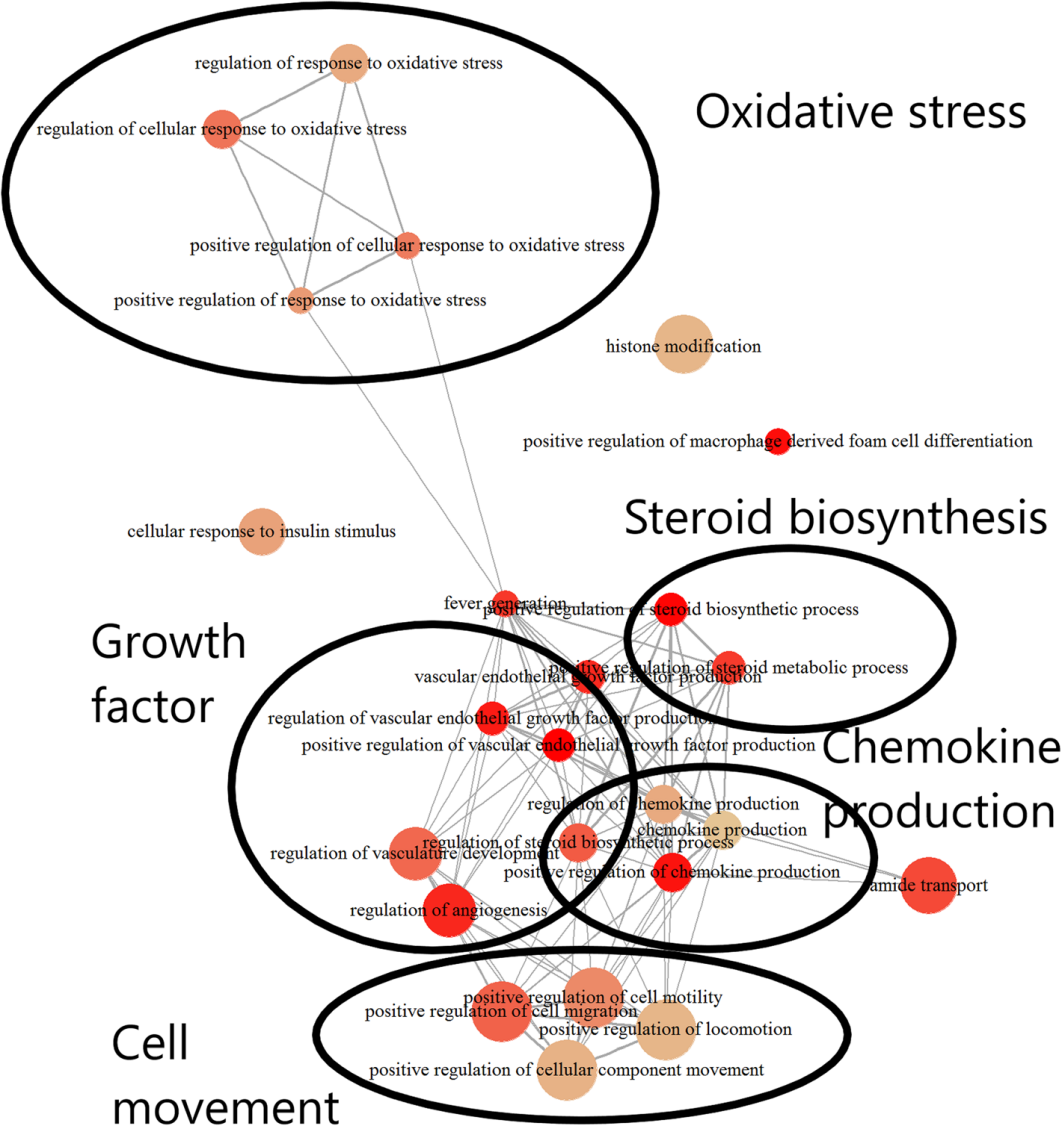
Frontal cortex RNA-seq significantly enriched gene ontology biological process annotations. Gene ontology biological process annotations significantly enriched in genes which were differentially expressed in the frontal cortex of CORT + DFP exposed mice, with groups of similar annotations highlighted

In the frontal cortex, the RNA-seq analysis identified 206 GENCODE genes (204 with unique entrez IDs) that were uniquely differentially expressed in the CORT + DFP exposure group compared to all other groups (Additional file 3: Table S1). Enrichment analysis showed 12 enriched KEGG pathways (Additional file 4: Figure S3; Additional file 5: Table S2) and 24 enriched GO BP annotations (Fig. 2; Additional file 6: Table S3). These annotations formed several broad groups related to immune response, including chemokine production, oxidative stress and steroid biosynthesis.

**Fig. 3:**
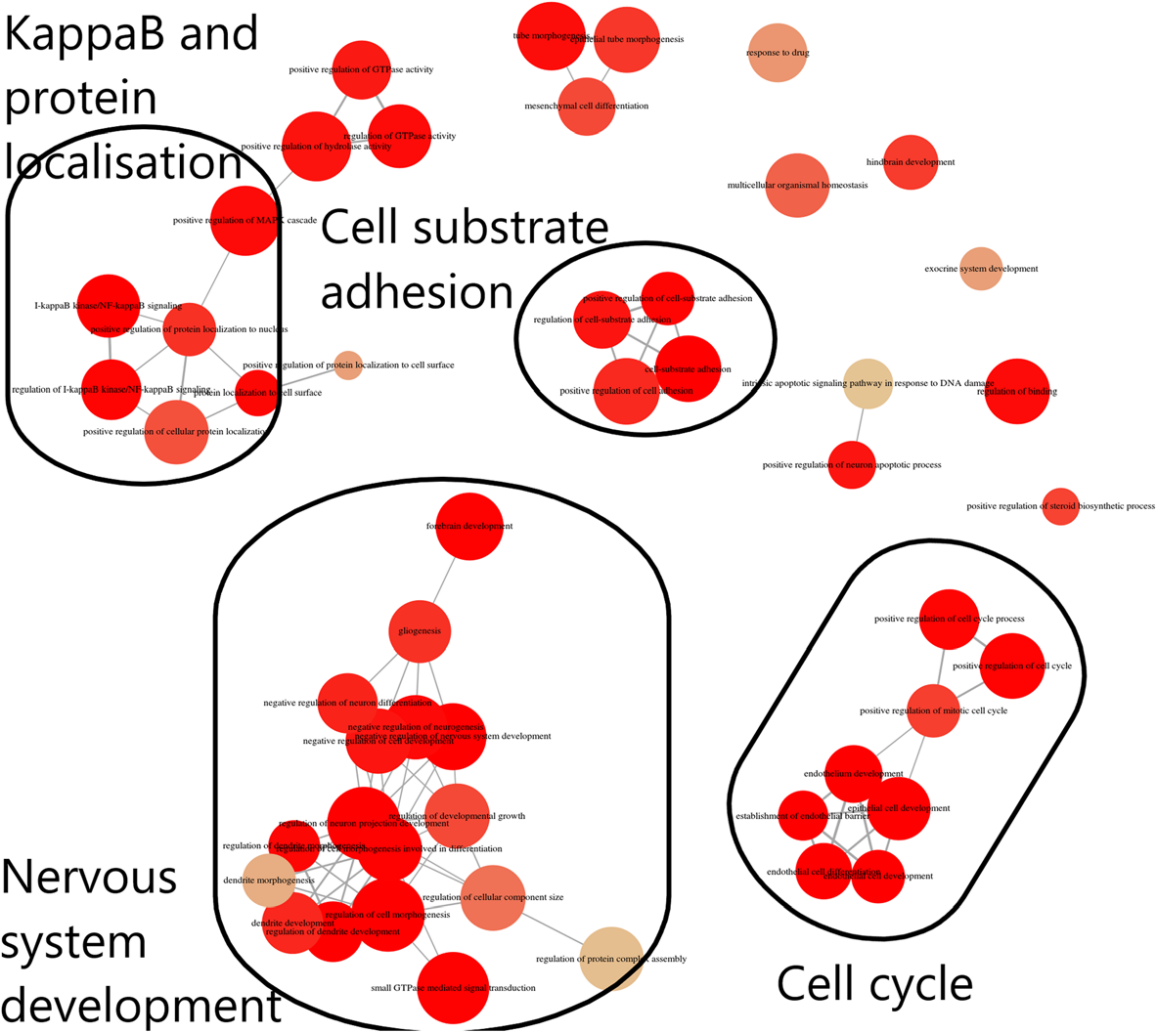
Hippocampus RNA-seq significantly enriched gene ontology biological process annotations. Top 50 gene ontology biological process annotations significantly enriched in genes that were differentially expressed in the hippocampus of CORT + DFP exposed mice, with groups of similar annotations highlighted

In the hippocampus, 667 GENCODE genes (637 with unique entrez IDs) were uniquely differentially expressed in the CORT + DFP exposure group (Additional file 7: Table S4) compared to all other groups. Enrichment analysis showed 19 enriched KEGG pathways (Additional file 8: Figure S4, Additional file 9: Table S5) and 294 enriched GO BP annotations (Fig. 3, Additional file 10: Table S6). Similar to the frontal cortex, these annotations were grouped into several clusters (Fig. 3), including immune-related annotations (e.g. I-kappaB and NF-kappaB signalling), annotations related to nervous system differentiation, and development.

The two analyses revealed two overlapping KEGG annotations: cytokine–cytokine receptor interaction and rheumatoid arthritis and nine GO BP annotations (Additional file 11: Table S7). There are 32 genes differentially expressed under CORT + DFP priming and exposure found in both the cortex and hippocampus RNA-seq data (Additional file 12: Figure S5; Additional file 13: Table S8).

Enrichment analysis of these 32 genes showed 31 enriched KEGG pathways, including annotations such as rheumatoid arthritis and cytokine–cytokine receptor interaction (Additional file 14: Table S9) and 333 GO BP annotations, including positive regulation of steroid biosynthetic process, positive regulation of chemokine production and regulation of I-kappaB kinase/NF-kappaB signaling (Additional file 15: Table S10).

### DNA methylation modifications

We next examined DNA methylation in the frontal cortex and hippocampus using RRBS to identify DNA methylation modifications associated with the exposures. The frontal cortex RRBS data showed 297 differentially methylated cytosines corresponding to 60 differentially methylated regions. Once these regions were annotated to genes (53 entrez IDs; 60 GENCODE; Additional file 16: Table S11), there was no significant enrichment for any KEGG or GO BP annotations. The hippocampus RRBS data showed 926 differentially methylated cytosines corresponding to 192 differentially methylated regions and annotated to 98 GENCODE genes (95 unique entrez IDs; Additional file 17: Table S12). Enrichment analysis was carried out for KEGG pathways or GO BP annotations, showing three significant GO BP enrichments: norepinephrine metabolic process (*n* = 3, *p* = 0.048, *q* = 0.045), cilium morphogenesis (*n* = 7, *p* = .048, *q* = 0.045) and cilium organization (*n* = 7, *p* = 0.049, *q* = 0.046). It is interesting to note in relation to the acetylcholinesterase action of DFP that a CpG site within the acetylcholinesterase gene (*Ache*) was significantly differentially methylated in the hippocampus (chr5: 137291317; adjp = 0.0296).

Given our hypothesis that DNA methylation modifications contribute to long-term changes in gene expression as a function of GWI exposures, this apparent lack of large, coordinated changes in DNA methylation was unexpected but could have at least two explanations. First, DNA methylation is thought to be relatively stable, and therefore, there may not have been an opportunity for substantial methylation changes to have occurred only 6 h after DFP exposure. A second possibility is that any changes were confounded by the number of different cell types within the brain. Consequently, methylation changes from any single cell type, especially cells making a small proportion of the tissue, may be lost in the ‘noise’. To investigate this second possibility, we used RNA-seq data to estimate the proportions of cells in our two tissues.

### Cell proportions

**Fig. 4:**
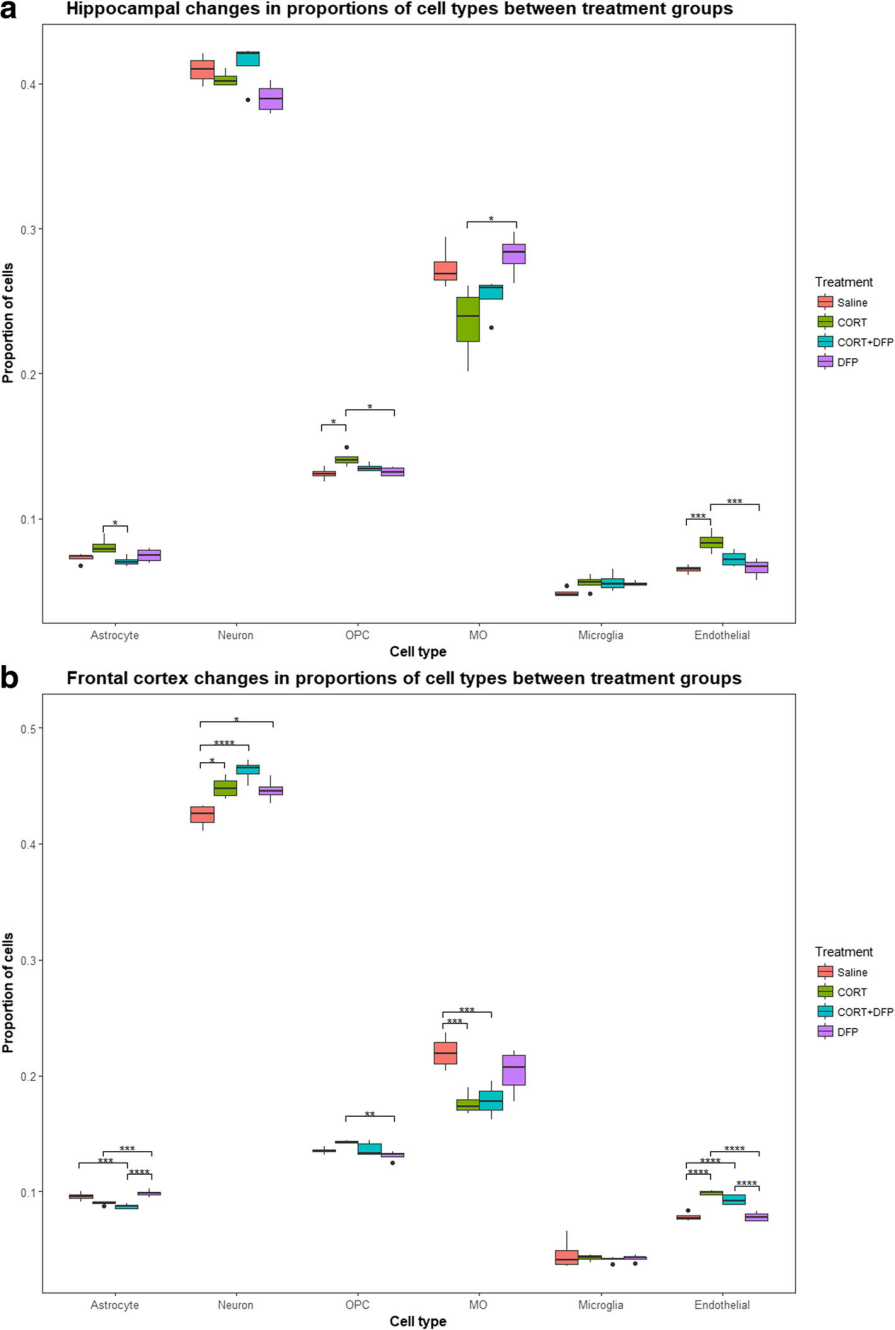
Estimated cell proportions from RNA-seq data. **a** Hippocampus and **b** frontal cortex cell proportions, estimated from RNA-seq data. Proportion of five cell types of interest in each exposure group, showing significant differences due to exposure. OPC oligodendrocyte precursor cells, MO myelinating oligodendrocytes. *p < 0.05, **p < 0.01, ***p < 0.005, ****p < 0.001

The estimated average proportion of each of our five cell types of interest for each exposure group is shown in Fig. 4. In the rat cortex, neurons make up ~ 40% of cells [74] and ~ 44% of whole mouse brain [75]. In the human and mouse cortex, microglia make up ~ 5% of cells [76, 77]. These reports are in line with our estimates of ~ 40–50% of cells being neurons and ~ 4–6% of cells being microglia. This, therefore, may indicate that enriching for specific cell types, such as microglia, may enhance our ability to detect cell-type-specific methylation modifications due to these exposures. For example, currently only ~ 1:25 RRBS counts will come from microglia.

An interesting incidental finding in the cortex was that CORT exposure, with or without co-exposure with DFP, was associated with an increase in the proportion of neurons and a decrease in the proportion of myelinating oligodendrocytes (MOs) in the frontal cortex (Fig. 4b). As we would not expect neurogenesis to occur in the frontal cortex, this suggests that the increase in the proportion of neurons is driven by a decrease in the absolute number of myelinating oligodendrocytes. A reduced number of oligodendrocytes would be in line with previous work in rats where CORT was shown to reduce the proliferation of oligodendrocytes [78, 79]. We emphasize that these are estimated cell proportions; however, the results indicate that stereology to confirm this will be important in future studies.

### Chromatin accessibility

H3K27ac is a mark of regions of the genome that are being actively transcribed [59, 60]. Our RNA-seq data showed enrichment for genes involved in histone modification, suggesting that changes in chromatin accessibility may play a role in the response to the exposures. H3K27ac ChIP-seq provides an additional layer of epigenetic regulation, which may respond more quickly than methylation. It also allows an indirect examination of current transcription in the largest cell population, as H3K27ac is found at actively transcribed regions. In ChIP-seq (and RRBS), every locus gives a single signal: either it is enriched for H3K27ac or it is not (or is methylation or is not). However, in RNA-seq, every locus could produce none, one or hundreds of RNA molecules, meaning that a small cell population with large changes in gene expression could mask the signal from a large population with small changes in gene expression. Therefore, using ChIP-seq will allow indirect examination of potential gene expression changes in neurons.

PePr identified 3294 GENCODE genes (3023 entrez IDs; Additional file 18: Table S13) with differential enrichment of H3K27ac, whereas diffReps identified 1518 GENCODE genes (1465 entrez IDs; Additional file 19: Table S14). The overlap between these two analyses was 563 GENCODE genes (557 entrez IDs; Additional file 20: Table S15) which were used for further analysis. However, gene annotation enrichment for each of the two gene sets (PePr and diffReps) demonstrated a large overlap in enriched annotations, suggesting that they are both finding changes in similar pathways but that the individual pathway members they find are different (85% of diffRep and 69% of PePr KEGG pathways (74) are found in both; 68% of diffReps and 54% of PePr GO BP annotations (521) are found in both).

**Fig. 5:**
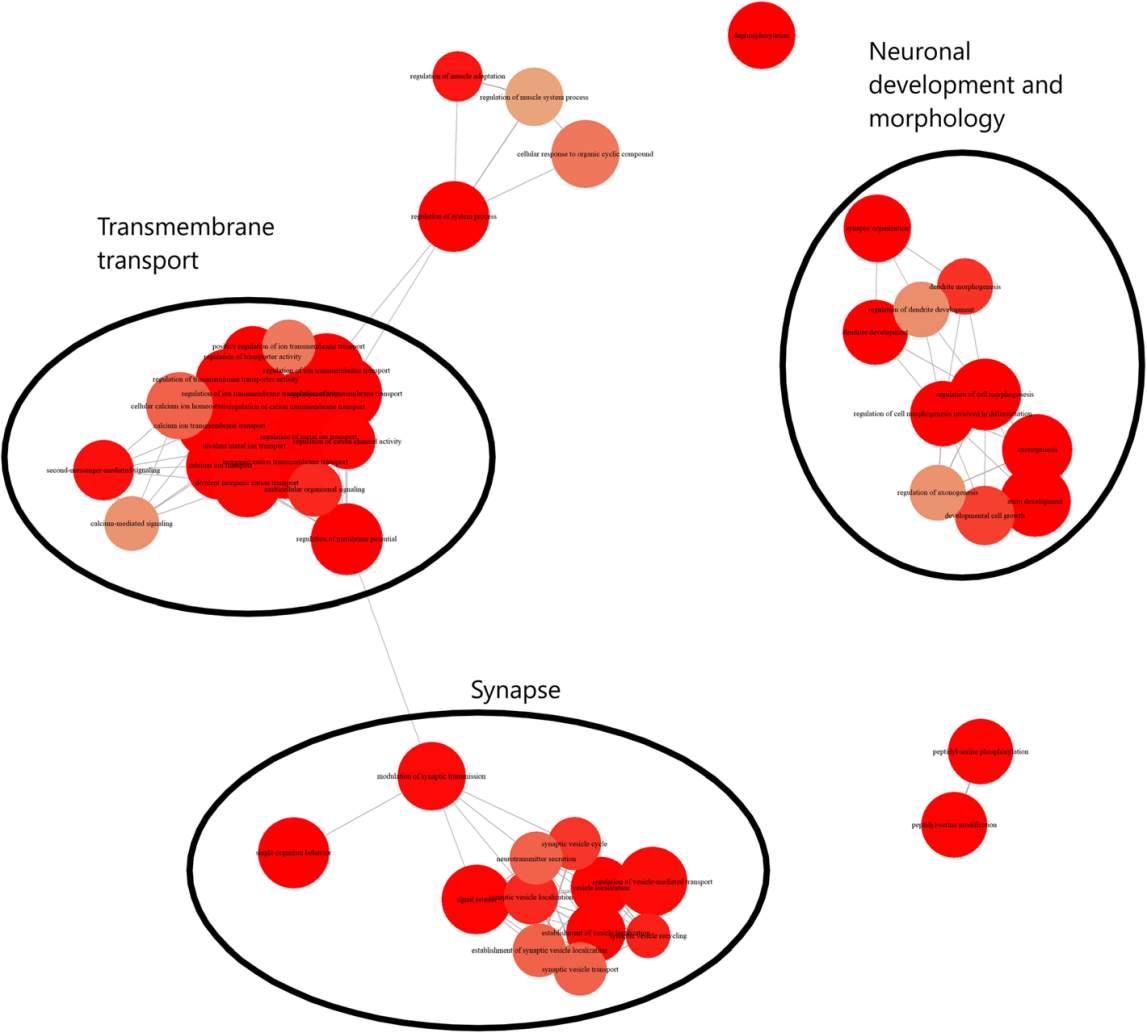
Frontal cortex H3K27ac ChIP-seq significantly enriched gene ontology biological process annotations. Top 50 GO BP annotations significantly enriched for differential enrichment of H3K27ac with CORT + DFP exposure

The enrichment data indicated a clear bias towards neuronal-linked annotations, including neuronal morphology and synapse-related annotations (Additional file 21: Figure S6 and Fig. 5; Additional file 22: Table S16 and Additional file 23: Table S17). Of particular interest are the observed enrichment of GO BP annotations ‘cognition’ and ‘learning or memory’, both of which are observed to be disrupted in GWI study participants, ‘Circadian entrainment’ which may relate to observed sleep disruption, and ‘response to steroid hormone’, which likely relates to CORT (a steroid hormone involved in the response to stress).

These findings demonstrate that there are potential changes in neuronal-related gene expression in the frontal cortex, as was also seen in the hippocampus RNA-seq, highlighted by the fact that 33 genes were found in both the ChIP-seq frontal cortex analysis and the hippocampus RNA-seq analysis.

### Overlap between genes found in different analyses

**Fig. 6:**
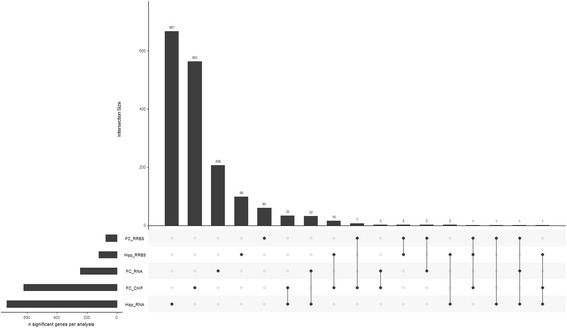
Annotated GENCODE genes found in each of our differential analyses. UpSetR diagram [73] of annotated GENCODE genes found in each of our differential analyses: frontal cortex RRBS (FC RRBS), frontal cortex H3K27ac ChIP-seq (FC ChIP), hippocampus RNA-seq (Hipp RNA), frontal cortex RNA-seq (FC RNA) and hippocampus RRBS (Hipp RRBS)

As shown in Fig. 6, there is not a large overlap in genes found between any of our analyses. However, this disparity may be partly explained from the aforementioned difference between mRNA and DNA, whereby one locus can produce many mRNA molecules, but DNA either has a modification or does not. This is reflected by the fact that the largest percentage overlap is between those genes found with RRBS and ChIP-seq, as these are both examining DNA modifications: 12% of genes found in frontal cortex RRBS, and 16% in hippocampus RRBS, are also found in the frontal cortex ChIP-seq, whereas this is only 1% and 5% for frontal cortex and hippocampus RNA-seq respectively. Similarly, 15.5% of genes found in the frontal cortex RNA-seq are also found in the hippocampus RNA-seq.

Because of the very few annotations enriched in genes differentially methylated in either of our tissues, we see very little overlap between annotations in methylation and ChIP-seq. However, we do see annotations and pathways enriched in both our ChIP-seq and RNA-seq data (Additional file 24: Figure S7 and Additional file 25: Figure S8).

## Discussion

To our knowledge, this is the first transcriptome- and epigenome-wide study to examine evidence for transcriptional, chromatin and DNA methylation modifications in a model of GWI. A previous study [12] examined 84 microRNAs (MiRNAs) and global but not gene-specific changes in DNA methylation and hydroxymethylation in rats subjected to restraint stress and a protocol of PB, DEET and permethrin to simulate troops’ chemical exposures. This study found differential expression of two miRNAs and in increase in global methylation in the hippocampus. Although this was an important step forward, our study was able to examine the whole transcriptome, a histone modification associated with chromatin accessibility and DNA methylation at a genome-wide, single cytosine level.

Overall, our results represent several interesting findings. First, as expected, there was a large change in the expression of immune-related genes in both the frontal cortex and hippocampus, building upon previous findings in this model [4]. Second, many genes associated with synaptic function are changed in their activity, as shown by our frontal cortex H3K27ac ChIP-seq data and hippocampus RNA-seq. These changes in gene expression seem to be subtler than those found for immune-related genes (lower expression), but differential expression of genes related to synaptic function in mice is associated with impaired memory and cognition, consistent with impairments reported by GWI suffers. Interestingly, long-term potentiation- and depression-related genes are enriched in the ChIP-seq data. Finally, we see evidence of not just a change in gene activity but of a suggested change in cell proportions. This is in line with previous work with CORT [78, 79].

It is possible that microglia are responsible for this large change in immune-related gene expression, but we acknowledge that other cells, such as astrocytes, also express cytokines and chemokines (e.g. Kim et al. [80]). However, this is usually at a much lower level than that in microglia: in genes significantly differentially expressed uniquely with CORT + DFP in both the frontal cortex and hippocampus the six genes with the largest fold change are expressed in microglia (Additional file 26: Table S18). Therefore, although other cell types may be contributing to cytokine and chemokine expression, it is highly unlikely that microglia are not the cell type driving this change. Similarly, we attribute many of the transmembrane transporter-related annotations we see in the frontal cortex ChIP-seq data and hippocampus RNA-seq data to neurons; however, many of these transporters are also expressed in glial cells [54]. For this reason, future work should be carried out to isolate or enrich specific cell populations, allowing these predictions to be tested.

Our findings of altered cholinergic neurotransmitter expression after DFP exposure are in line with previous studies of low dose sarin exposure in rats [81, 82]. Specifically, changes in M1 and M3 acetylcholine receptor expression were seen in the frontal cortex in a dose-dependent manner when the animals were maintained under hyper-thermic conditions (i.e. a stressor; Henderson et al. [81, 82]). In relation to this, we see choline-related annotations at all levels we analysed: our H3K27ac ChIP-seq analyses show changes in cholinergic synapse-related genes and differential binding related to *Chrm2*, the gene coding for M2; the KEGG annotation, ‘choline metabolism in cancer’, is enriched in our hippocampus RNA-seq genes and our H3K27ac ChIP-seq genes; finally, in our hippocampus RRBS, we see altered methylation of a CpG site within *Ache*, the gene coding for acetylcholinesterase.

These findings not only link to rat models but also to GWI subjects, where differences in prefrontal cortex working memory have been previously found and have been attributed to the cholinergic system [83]. This again connects with our findings from ChIP-seq where both ‘learning and memory’ and ‘cholinergic synapse’ annotations are significantly enriched. Therefore, pre-exposure with CORT may not only be potentiating the immune response to AchE inhibitors but also causing longer term changes to the cholinergic signalling system in line with previous animal models. Human studies have been more varied, with evidence both for [84, 85] and against [86] long-term changes in the cholinergic system, perhaps related to the heterogeneity of symptoms or to differences in tissues being examined. Recent work by Locker et al. [15] has shown that increased neuroinflammatory response in this model is not directly related to AChE inhibition but instead may result from changes in other aspects of signaling (e.g. epigenetic alterations in gene expression).

In relation to the potential reduction in myelinating oligodendrocytes in the cortex, this may have an effect on some of the phenotypes seen in GWI: reduced oligodendrocytes have been linked to major depressive disorder (MDD), functional consequences in neurons and mood-related symptoms in rats [87]. As this change in cell proportion would affect myelinating cells, it could also contribute to the reported alterations in white matter in GWI veterans [20, 88–90].

Our RNA-seq data suggests both immune dysfunction and oxidative stress. Previous reports have shown immune dysfunction and oxidative stress in other disorders with similar phenotypes to GWI such as myalgic encephalomyelitis/chronic fatigue syndrome (ME/CFS) [91]. However, whether the immune response is causing oxidative stress or oxidative stress is causing an immune response is less clear. This is directly relevant, as oxidative stress contributes to the toxicity of AchE inhibitors [29] and has been suggested as a cause of GWI [1, 92].

One of the six genes (Additional file 26: Table S18) we find strongly differentially expressed in both the frontal cortex and hippocampus, *Tlr2*, has previously been linked to sickness behaviour when expressed in hypothalamic microglia [93] and was reported to show increased expression in a model of GWI [94]. Therefore, it is of great interest in terms of the sickness-like behaviour that defines GWI. TLR activation by immune insult appears to increase *Tlr2* expression [95]. We can speculate that the change in *Tlr2* expression we see is upstream of the change in *Il1b* and *TNF*, as specific activation of TLR2 increases their expression [93, 96]. Further, TLR stimulation increases *SLC15A3* expression in dendritic cells, which in turn regulates *TNF* and *IL-1β* expression [97]. At the very least, these six, consistently, strongly upregulated genes make up a robust core of immune- and microglia-related genes for further investigation (Additional file 26: Table S18).

Our results correspond very well with those of Broderick et al. [98] in blood samples from GWI study participants, who found changes to NF-KappaB-related genes (which are enriched in our hippocampus RNA-seq data and in the genes significant in both the cortex and hippocampus RNA-seq), and in pathways under the broad theme of neuronal development and migration, which we see in our hippocampus RNA-seq and our cortex ChIP-seq. They also saw ligand–receptor interactions supporting neurotransmission, which again we see strongly in our cortex ChIP-seq data. Further, our KEGG pathway analysis of the transcriptomics data highlighted rheumatoid arthritis-related gene enrichment. Interestingly, a study in veterans with GWI also highlighted the possibility of medication used to treat rheumatoid arthritis also being used to treat GWI [99]. The same study identified the TNF-alpha pathway (which we see in our transcriptomics) and the estrogen pathway (which we saw in our ChIP-seq) as potential drug targets [99]. Therefore, data from our model corresponds to data identified from veterans with GWI and could be used as a model to test these compounds as potential therapeutics.

A recent paper described eight potential blood biomarkers for GWI, the strongest being a 9.27-fold increase in CaMKII protein in the blood of these veterans [100]. In our study, *Camk2b* was found to be differentially methylated in the hippocampus and have differential H3K27ac in the cortex.

Further linking our study with human data was the finding that plasma CRP levels are increased in the blood of GWI veterans [38]. CRP is often used as a biomarker of IL-6-mediated inflammation [38, 101], and IL-6 related annotations were enriched in the genes differentially expressed in both the cortex and hippocampus (Additional file 15: Table S10). This annotation was due to four genes, *Il1b*, *Tlr2*, *Il1a* and *Tnf*, four of our six most strongly and consistently differentially expressed genes.

We see minimal overlap in genes with significant differential methylation between the hippocampus and frontal cortex (two genes, *Bcar3* and *Tmem242*). This is likely due, at least partly, to the two factors outlined above: cellular heterozygosity and a short time point after treatment. Of the two consistent genes, *Bcar3* is involved in cell proliferation, which when overexpressed in breast cancer conveys estrogen resistance, and *Tmem242* is a transmembrane protein with very little current annotation. We cannot detect any coordinated methylation changes (denoted by enriched annotations in our significant genes) in the frontal cortex and very few in the hippocampus. As mentioned above, we detect differential methylation of one CpG site within *Ache*, the gene coding for acetylcholinesterase. A number of the genes found to be differentially methylated can be linked to GWI. Examples include *Lims1*, which is known to be regulated by TNF and is involved in cell growth and survival [102]; *Sesn1*, *Aplnr*, *Pxn* and *Actn1*, which have been linked to ME/CFS [103, 104]; *Col5a3*, which was identified in a rat model of GWI in the hippocampus [12]; and *Slc1a2*, which has been linked to ALS, a disorder GW veterans are at increased risk of [105, 106]. This may indicate that networks of genes linked to GWI are only just beginning to be methylated, or that different groups of genes are methylated in different cell types. However, until these coordinated networks are elucidated, investigation of individual genes could lead to spurious associations.

## Conclusions

We are able to show a range of changes in the transcriptome of this well-established mouse model, many of which reflect gene expression seen in veterans with GWI. Further, we see alteration in H3K27ac, showing potential chromatin configuration changes, which could lead to epigenetic effects with long-lasting implications. We also find differences in DNA methylation, although these are less easily interpretable than the transcriptome and H3K27ac changes.

Additional research is needed to assess whether effects of these epigenetic and transcriptional modifications on long-term health outcomes are cumulative and/or are potentiated by later exposures (e.g. infection). Notably, however, our findings reveal gene pathways known to be involved in long-term adverse health effects in GWI veterans. These results suggest that epigenetic and transcriptional regulation during the initial exposure period likely contribute to pathological outcomes in GWI. It would be important to examine these modifications in peripheral tissues from GWI veterans to ascertain whether biomarkers could be developed to predict future health outcomes.

## Additional files

Additional file 1: **Figure S1.** Poisson dissimilarity matrix for hippocampus RNA-seq. Heatmaps showing the Poisson dissimilarity matrix for our hippocampus RNA-seq samples. Darker blue indicates more similar samples. (CSV 52 kb)

Additional file 2: **Figure S2.** Poisson dissimilarity matrix from frontal cortex RNA-seq. Heatmaps showing the Poisson dissimilarity matrix for our frontal cortex (B) RNA-seq samples. Darker blue indicates more similar samples. (CSV 2 kb)

Additional file 3: **Table S1.** Genes identified as uniquely differentially expressed in the frontal cortex after CORT + DFP exposure, compared to all other groups. (TIFF 2391 kb)

Additional file 4: **Figure S3.** Frontal cortex RNA-seq significantly enriched KEGG pathway annotations. KEGG pathways significantly enriched in genes that were differentially expressed in the frontal cortex of CORT + DFP exposed mice. (CSV 6 kb)

Additional file 5: **Table S2.** KEGG pathways enriched in the genes identified as uniquely differentially expressed in the frontal cortex after CORT + DFP exposure, compared to all other groups. (CSV 4 kb)

Additional file 6: **Table S3.** Gene Ontology (GO) Biological Process (BP) annotations enriched in the genes identified as uniquely differentially expressed in the frontal cortex after CORT + DFP exposure, compared to all other groups. (CSV 38 kb)

Additional file 7: **Table S4.** Genes identified as uniquely differentially expressed in the hippocampus after CORT + DFP exposure, compared to all other groups. (CSV 1 kb)

Additional file 8: **Figure S4.** Hippocampus RNA-seq significantly enriched KEGG pathway annotations. KEGG pathways significantly enriched in genes that were differentially expressed in hippocampus of CORT + DFP exposed mice. (CSV 3 kb)

Additional file 9: **Table S5.** KEGG pathways enriched in the genes identified as uniquely differentially expressed in the hippocampus after CORT + DFP exposure, compared to all other groups. (CSV 103 kb)

Additional file 10: **Table S6.** Gene Ontology (GO) Biological Process (BP) annotations enriched in the genes identified as uniquely differentially expressed in the hippocampus after CORT + DFP exposure, compared to all other groups. (CSV 55 kb)

Additional file 11: **Table S7.** Gene Ontology (GO) Biological Process (BP) annotations found to be enriched in genes significant in the frontal cortex (Additional file 6: Table S3) and enriched in genes significant in the hippocampus (Additional file 10: Table S6). (TIFF 1648 kb)

Additional file 12: **Figure S5.** Overlap of genes significantly differentially expressed with CORT + DFP exposure in the frontal cortex and hippocampus. (CSV 18 kb)

Additional file 13: **Table S8.** Genes identified as uniquely differentially expressed in both the frontal cortex (Additional file 3: Table S1) and the hippocampus (Additional file 7: Table S4) after CORT + DFP exposure, compared to all other groups. (TIFF 29059 kb)

Additional file 14: **Table S9.** KEGG pathways found to be enriched in genes identified as uniquely differentially expressed in both the frontal cortex (Additional file 3: Table S1) and the hippocampus (Additional file 7: Table S4) after CORT + DFP exposure, compared to all other groups. (CSV 67 kb)

Additional file 15: **Table S10.** Gene Ontology (GO) Biological Process (BP) annotations found to be enriched in genes identified as uniquely differentially expressed in both the frontal cortex (Additional file 3: Table S1) and the hippocampus (Additional file 7: Table S4) after CORT + DFP exposure, compared to all other groups. (CSV 12 kb)

Additional file 16: **Table S11.** Genes identified as containing differentially methylated regions in the frontal cortex of combined CORT + DFP exposed animals. (TIFF 3010 kb)

Additional file 17: **Table S12.** Genes identified as containing differentially methylated regions in the hippocampus of combined CORT + DFP exposed animals. (TIFF 3010 kb)

Additional file 18: **Table S13.** Genes identified by PePr as having differential enrichment of H3K27ac in the frontal cortex of CORT + DFP exposed animals. (CSV 519 bytes)

Additional file 19: **Table S14.** Genes identified by diffReps as having differential enrichment of H3K27ac in the frontal cortex of CORT + DFP exposed animals. (TIFF 2386 kb)

Additional file 20: **Table S15.** Genes identified by both PePr and diffReps as having differential enrichment of H3K27ac in the frontal cortex of CORT + DFP exposed animals. (CSV 26 kb)

Additional file 21: **Figure S6.** Frontal cortex H3K27ac ChIP-seq significantly enriched KEGG pathways. Top 50 KEGG pathways significantly enriched for differential enrichment of H3K27ac with CORT + DFP exposure. (TIFF 540 kb)

Additional file 22: **Table S16.** Gene Ontology (GO) Biological Process (BP) annotations found to be enriched in genes identified as uniquely differentially enrichment H3K27ac in by both PePr and diffReps (Additional file 20: Table S15). (CSV 1 kb)

Additional file 23: **Table S17.** KEGG pathways found to be enriched in genes identified as uniquely differentially enrichment H3K27ac in by both PePr and diffReps (Additional file 20: Table S15). (CSV 3 kb)

Additional file 24: **Figure S7.** Comparison of significantly enriched gene ontology biological process annotations in each of our analyses. UpSetR diagram [73] of Gene Ontology (GO) biological process (BP) annotations enriched in genes significant in each of our differential analyses: frontal cortex RRBS (FC RRBS), frontal cortex H3K27ac ChIP-seq (FC ChIP), hippocampus RNA-seq (Hipp RNA), frontal cortex RNA-seq (FC RNA) and hippocampus RRBS (Hipp RRBS). (CSV 79 kb)

Additional file 25: **Figure S8.** Comparison of significantly enriched KEGG pathway annotations in each of our analyses. UpSetR [73] diagram of KEGG pathways enriched in genes significant in each of our differential analyses: frontal cortex RRBS (FC RRBS), frontal cortex H3K27ac ChIP-seq (FC ChIP), hippocampus RNA-seq (Hipp RNA), frontal cortex RNA-seq (FC RNA) and hippocampus RRBS (Hipp RRBS). (TIFF 317 kb)

Additional file 26: **Table S18.** Genes which are significantly differentially expressed uniquely in CORT + DFP and have a greater than 0.75-fold change in expression between saline and CORT + DFP in both frontal cortex and hippocampus. All six show an increase in expression. (CSV 3 kb)

## Abbreviations

AchE: Acetylcholinesterase
ChIP: Chromatin immunoprecipitation
ChIP-seq: Native chromatin immunoprecipitation sequencing
CORT: Corticosterone
CRP: C-reactive protein
DEET: *N*,*N*-Diethyl-meta-toluamide
DFP: Diisopropyl fluorophosphate
DMCs: Differentially methylated cytosines
DMRs: Differentially methylated regions
DNA: Deoxyribonucleic acid
DOSE: Disease Ontology Semantic and Enrichment
GEO: Gene Expression Omnibus
GO BP: Gene Ontology Biological Process
GWI: Gulf War illnesses
KEGG: Kyoto Encyclopedia of Genes and Genomes
ME/CFS: Myalgic encephalomyelitis/chronic fatigue syndrome
MNase: Micrococcal nuclease
MO: Myelinating oligodendrocytes
OPC: Oligodendrocyte precursor cells
PB: Pyridostigmine bromide
PBMCs: Peripheral blood mononuclear cells
RNA: Ribonucleic acid
RNA-seq: Ribonucleic acid sequencing
RRBS: Reduced representation bisulfite sequencing
TNF: Tumor necrosis factor
