# Supplementary figures and images for: Epigenetic impacts of stress priming of the neuroinflammatory response to sarin surrogate in mice: a model of Gulf War illness
Source: J Neuroinflammation. 2018 Mar 17;15:86. doi: 10.1186/s12974-018-1113-9 (PMC5857314; doi:10.1186/s12974-018-1113-9)

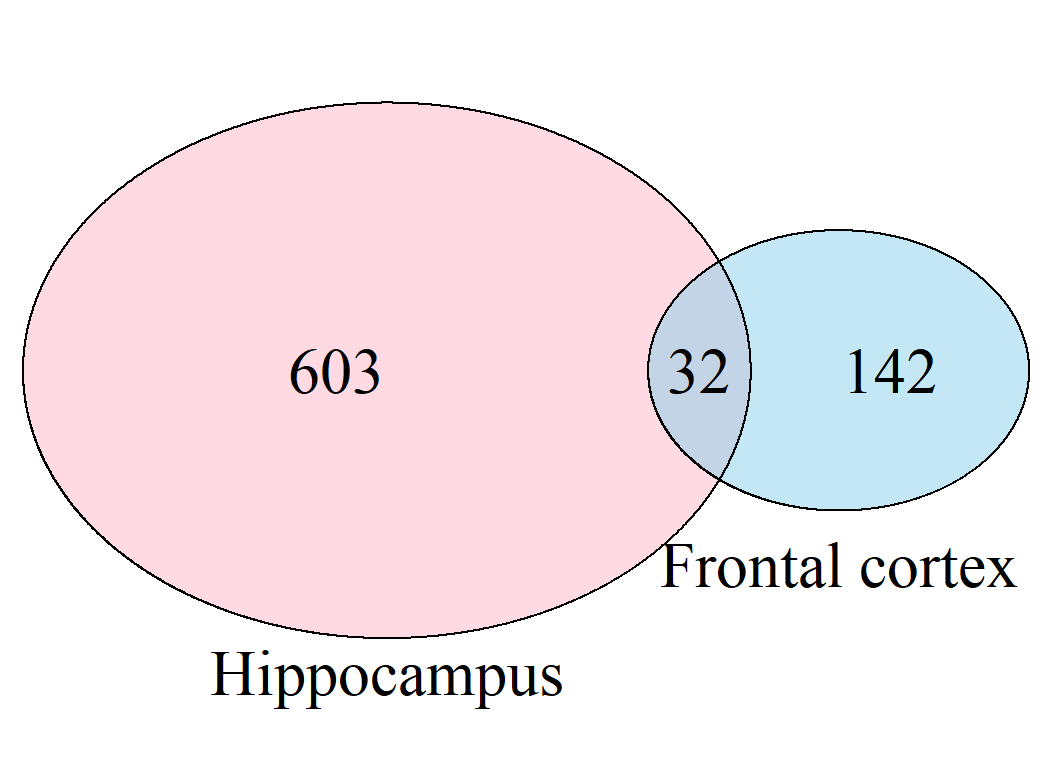

Supplement: Supplementary file 3 — Table S1. Genes identified as uniquely differentially expressed in the frontal cortex after CORT + DFP exposure, compared to all other groups. (TIFF 2391 kb) [file 12974_2018_1113_MOESM12_ESM.tiff]

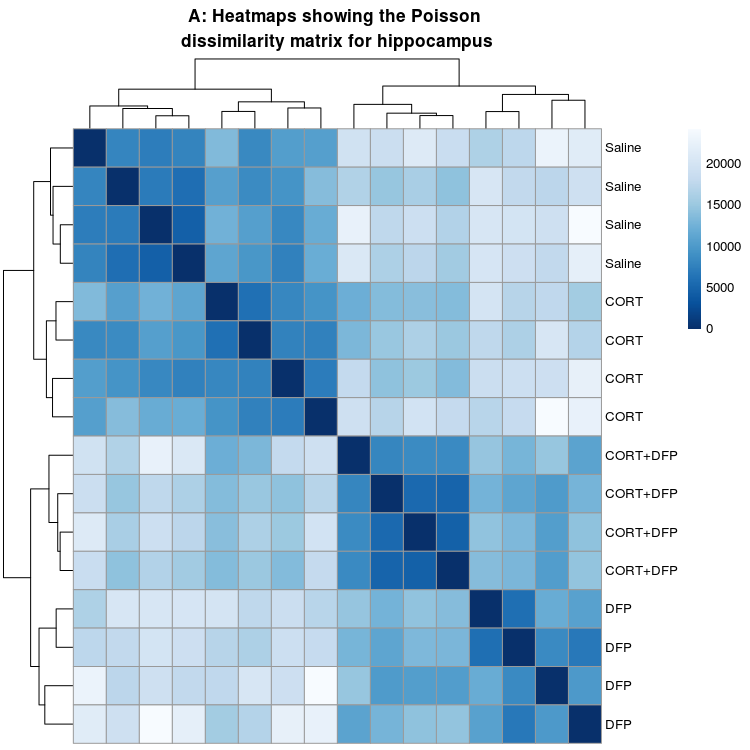

Supplement: Supplementary file 11 — Table S7. Gene Ontology (GO) Biological Process (BP) annotations found to be enriched in genes significant in the frontal cortex (Additional file 6: Table S3) and enriched in genes significant in the hippocampus (Additional file 10: Table S6). (TIFF 1648 kb) [file 12974_2018_1113_MOESM1_ESM.tiff]

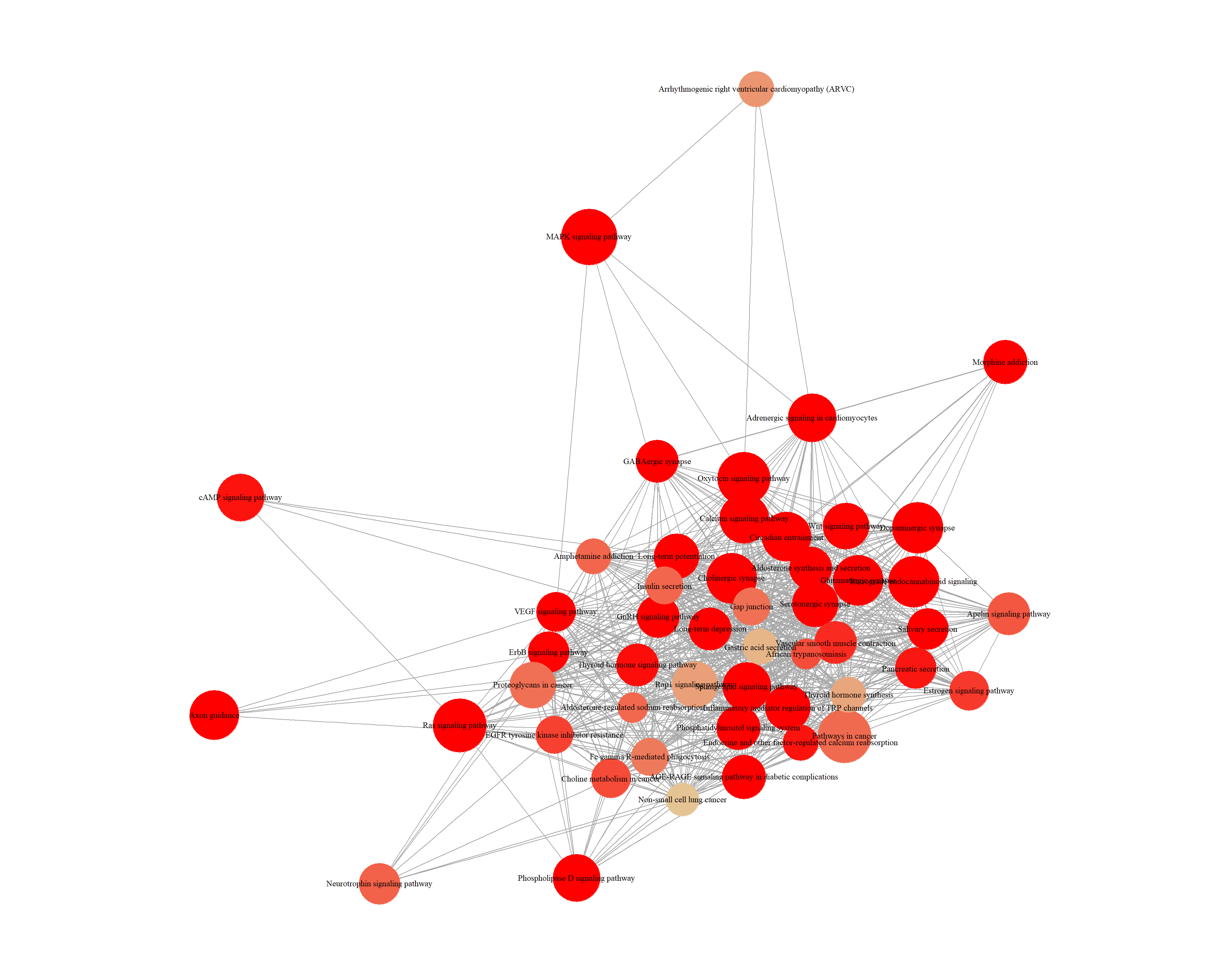

Supplement: Supplementary file 13 — Table S8. Genes identified as uniquely differentially expressed in both the frontal cortex (Additional file 3: Table S1) and the hippocampus (Additional file 7: Table S4) after CORT + DFP exposure, compared to all other groups. (TIFF 29059 kb) [file 12974_2018_1113_MOESM21_ESM.tiff]

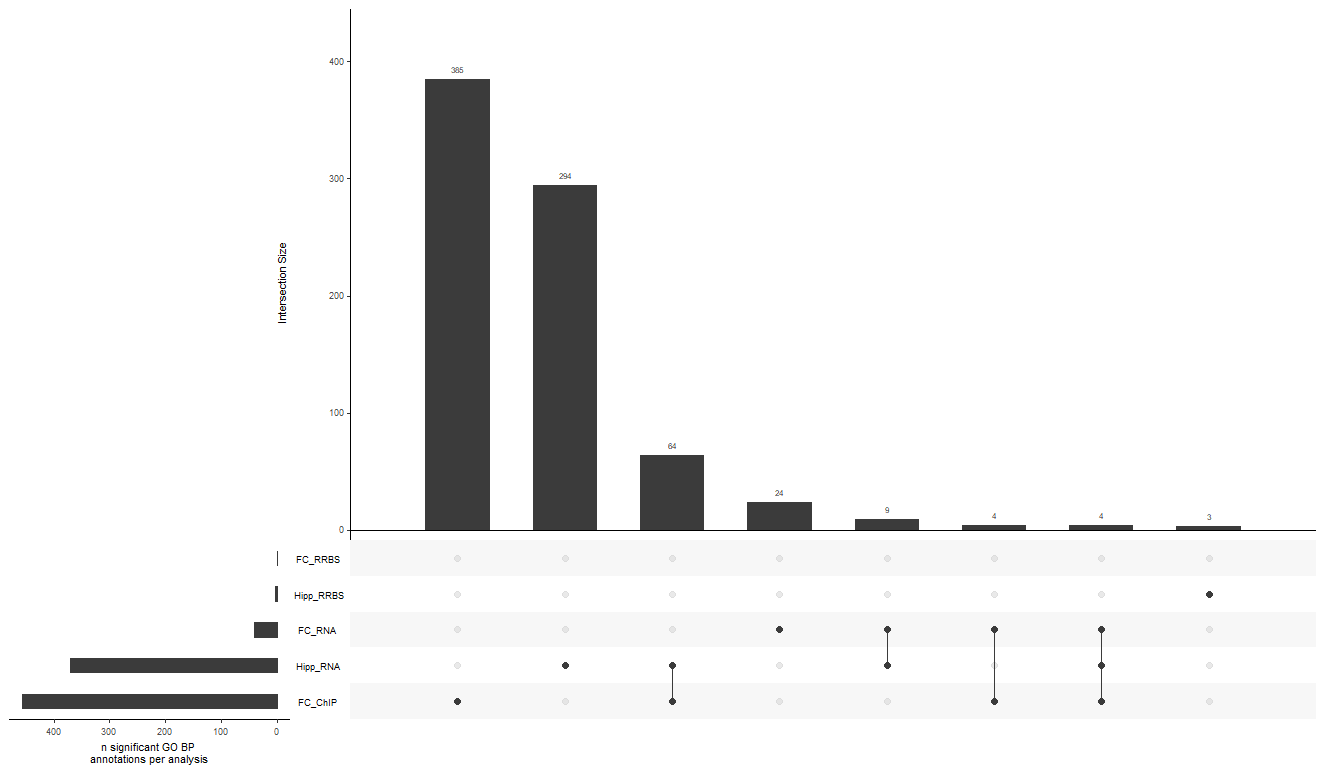

Supplement: Supplementary file 16 — Table S11. Genes identified as containing differentially methylated regions in the frontal cortex of combined CORT + DFP exposed animals. (TIFF 3010 kb) [file 12974_2018_1113_MOESM24_ESM.tiff]

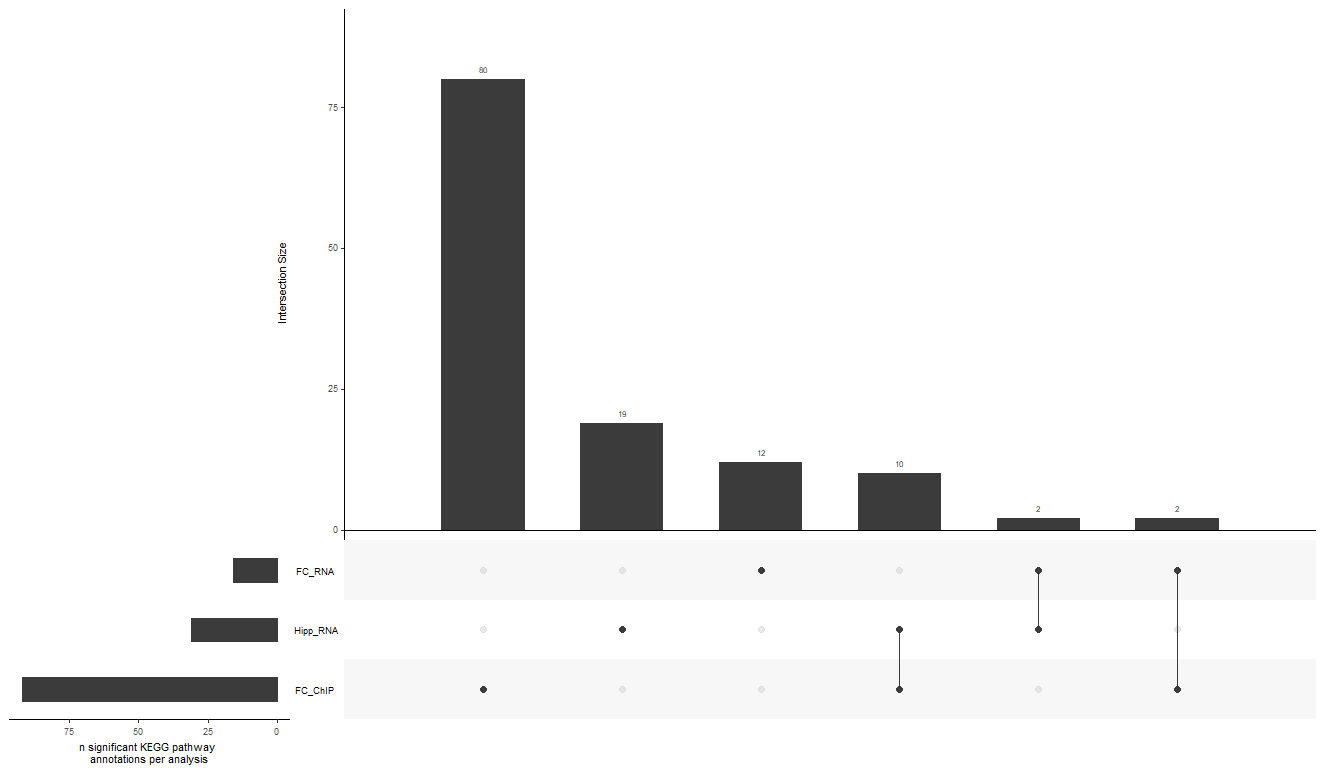

Supplement: Supplementary file 17 — Table S12. Genes identified as containing differentially methylated regions in the hippocampus of combined CORT + DFP exposed animals. (TIFF 3010 kb) [file 12974_2018_1113_MOESM25_ESM.tiff]

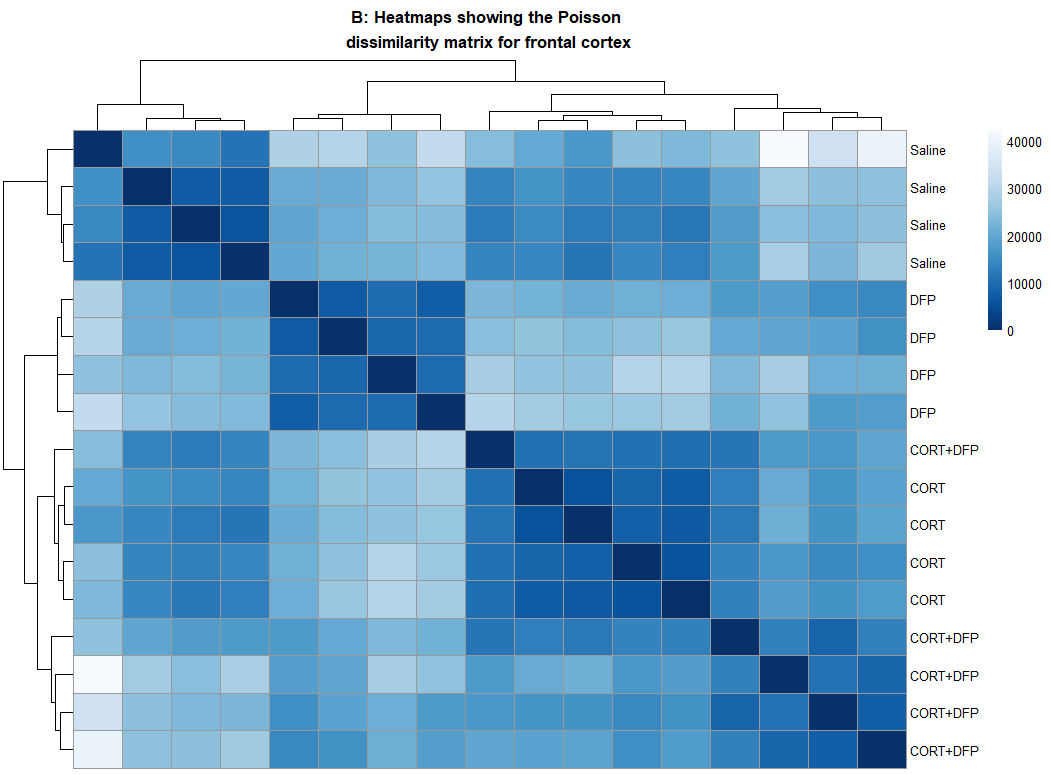

Supplement: Supplementary file 19 — Table S14. Genes identified by diffReps as having differential enrichment of H3K27ac in the frontal cortex of CORT + DFP exposed animals. (TIFF 2386 kb) [file 12974_2018_1113_MOESM2_ESM.tiff]

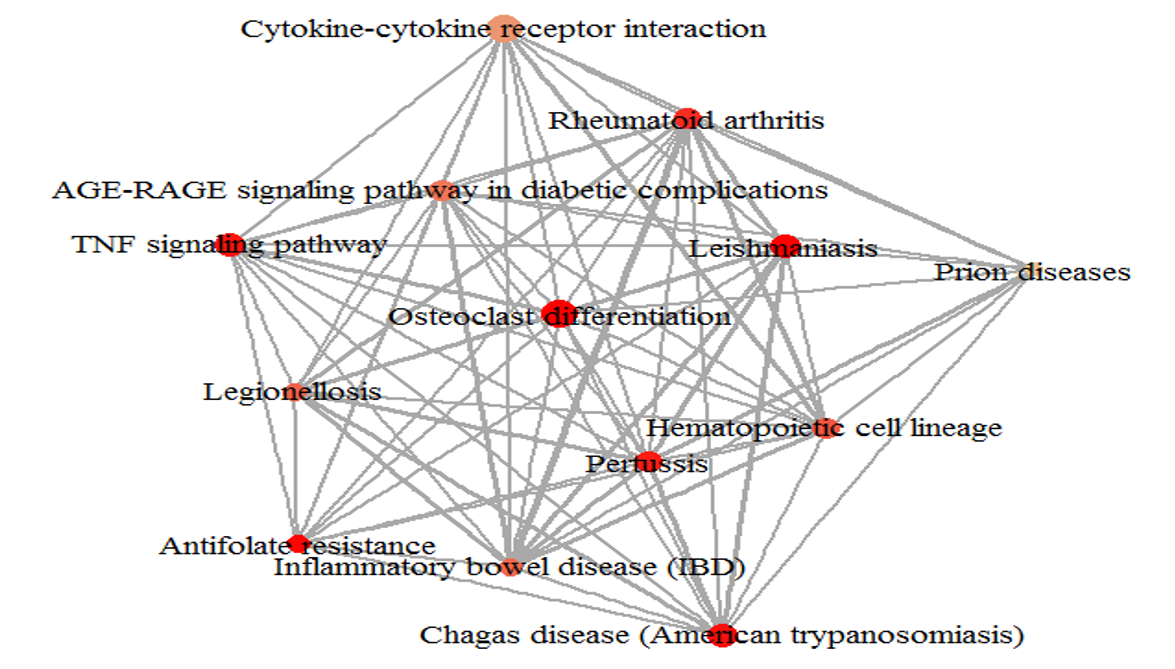

Supplement: Supplementary file 21 — Figure S6. Frontal cortex H3K27ac ChIP-seq significantly enriched KEGG pathways. Top 50 KEGG pathways significantly enriched for differential enrichment of H3K27ac with CORT + DFP exposure. (TIFF 540 kb) [file 12974_2018_1113_MOESM4_ESM.tif]

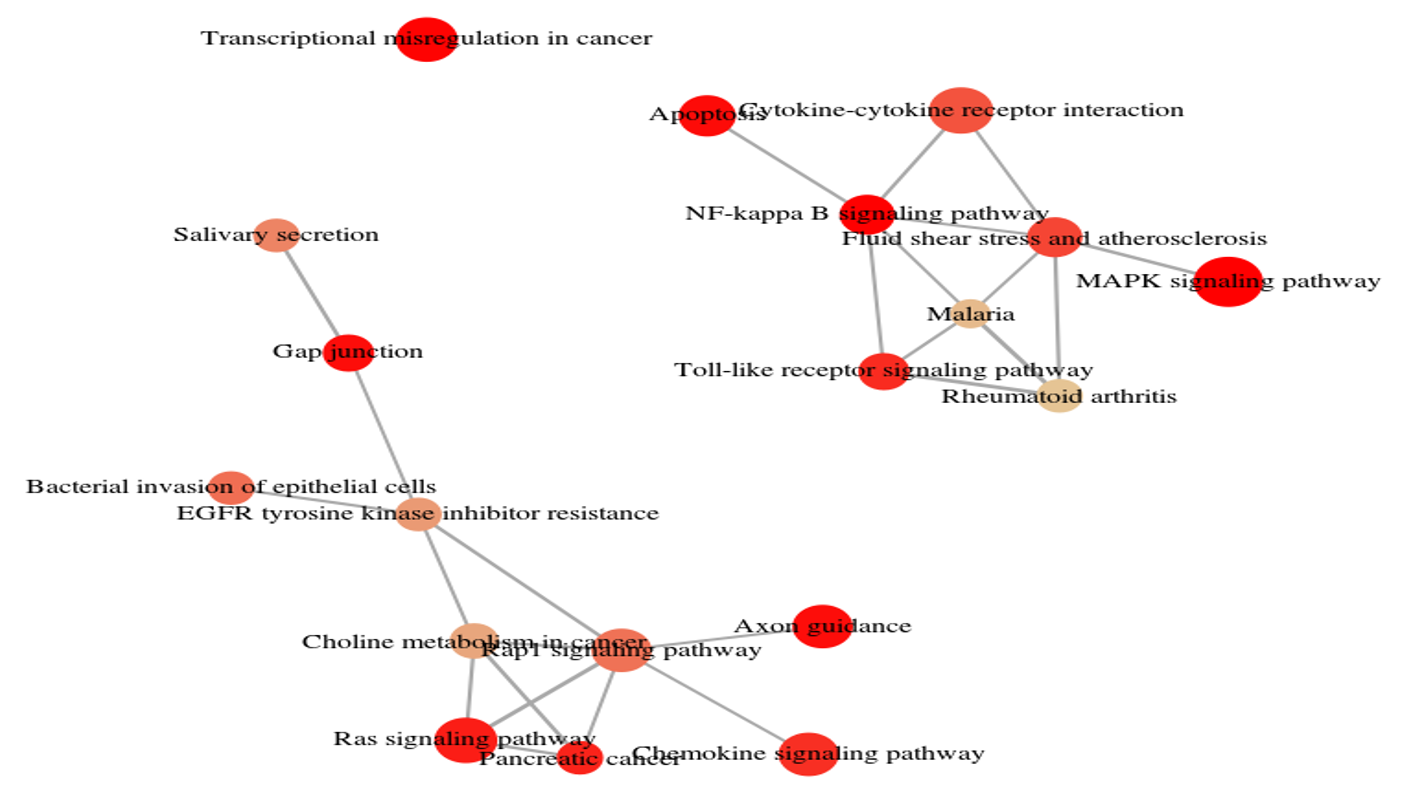

Supplement: Supplementary file 25 — Figure S8. Comparison of significantly enriched KEGG pathway annotations in each of our analyses. UpSetR [73] diagram of KEGG pathways enriched in genes significant in each of our differential analyses: frontal cortex RRBS (FC RRBS), frontal cortex H3K27ac ChIP-seq (FC ChIP), hippocampus RNA-seq (Hipp RNA), frontal cortex RNA-seq (FC RNA) and hippocampus RRBS (Hipp RRBS). (TIFF 317 kb) [file 12974_2018_1113_MOESM8_ESM.tif]
